# MRI-based SIR quantitative biomarkers: a novel imaging diagnostic strategy for thyroid eye disease activity staging

**DOI:** 10.3389/fendo.2025.1650116

**Published:** 2025-09-12

**Authors:** Muhan Cai, Jiani Yang, Xuemei Li, Ying Hu, Hongfei Liao, Chao Xiong

**Affiliations:** ^1^ School of Ophthalmology and Optometry, Jiangxi Medical College, Nanchang University, Nanchang, China; ^2^ Department of Ophthalmology, Affiliated Eye Hospital of Nanchang University, Nanchang University, Nanchang, China

**Keywords:** thyroid eye disease, magnetic resonance imaging, signal intensity ratio, activity, diagnosis

## Abstract

**Purpose:**

To evaluate the diagnostic efficacy of magnetic resonance imaging (MRI)-based signal intensity ratio (SIR) between extraocular muscles and white matter/temporal muscle for staging thyroid eye disease (TED) activity, and to provide a novel approach for diagnosis in active TED patients.

**Methods:**

A number of 40 patients with TED (79 eyes) and 65 controls (65 eyes) were recruited. MRI-based parameters of four extraocular muscles, ipsilateral white matter, temporal muscle, and other clinical factors were retrospectively collected. Patients were grouped according to disease activity determined by the Clinical Activity Score (CAS), and intergroup analysis was subsequently performed based on this classification. The signal intensities were measured using fat-suppressed T2-weighted imaging (T2WI-FS) sequences from MRI. The SIR of the extraocular rectus muscle to that of the ipsilateral white matter represents SIR1, while the SIR of the extraocular rectus muscle to that of the ipsilateral temporalis muscle represents SIR2.

**Results:**

Active TED group demonstrated elevated SIR1 and SIR2 values across all rectus muscles compared to control group and inactive TED group (P<0.05), with no differences between inactive TED and controls. Receiver operator characteristic (ROC) curve analysis identified SIR2 as superior to SIR1 for most muscles, with inferior rectus SIR1 achieving the highest AUC (0.837). Based on 95% confidence intervals and cutoff values, we propose redefining TED staging: control ranges (0.936–1.019) as absolute inactive phase, active TED ranges (1.210–1.344) as absolute active phase, and transitional values (1.019–1.210) as clinical vigilance phase requiring heightened attention. The model suggests that patients currently defined as “inactive” by CAS may have subclinical inflammation, explaining paradoxical disease progression in some cases.

**Conclusions:**

The signal intensity ratio (SIR) from fat-suppressed T2-weighted (T2WI-FS) sequences serves as a reliable predictor for TED activity. The 95% confidence interval (CI) for SIR values can provide a new strategy for early diagnosis.

## Introduction

Thyroid eye disease (TED), also known as Graves’ ophthalmopathy (GO) or thyroid-associated ophthalmopathy (TAO), is an autoimmune orbital disorder closely linked to thyroid dysfunction. It represents the most prevalent orbital disease in adults (1). TED is most frequently observed in patients with hyperthyroidism, serving as the predominant extra-thyroidal manifestation of Graves’ disease (GD). However, it may also occur in a minority of euthyroid individuals and those with hypothyroidism. The primary ocular manifestations include eyelid retraction, exophthalmos, strabismus, diplopia, and impaired ocular motility. In severe cases, patients may develop exposure keratitis, while compression of the optic nerve can lead to progressive vision loss and even blindness.

The natural progression of TED is characterized by an initial active phase (inflammatory stage) followed by a late inactive phase (fibrotic stage), with risks of recurrence and chronic sequelae (2, 3). Although orbital muscle biopsy is the gold standard for assessing TED activity, its invasive nature limits clinical application. The Clinical Activity Score (CAS) proposed by Mourits et al. in 1989 has become the primary clinical tool for evaluating TED activity (4, 5). However, CAS assessments are susceptible to subjective interference by doctors and exhibit limited comparability across ethnic groups due to anatomical variations in orbital structures. Furthermore, CAS focuses on anterior orbital features and fails to detect inflammation in deep orbital tissues. As appearance of patients often lag behind actual inflammatory status, a low score cannot rule out the inflammatory state of the patient’s orbital tissue (5). Overall, diagnosis using CAS score alone may cause missed diagnosis in some patients in early active stages.

Establishing objective biomarkers to define TED stages, particularly those can reflect deep orbital inflammation, is critical for timely disease management. Magnetic resonance imaging (MRI), with its superior soft-tissue resolution, has been widely adopted in TED diagnosis. The fat-suppressed T2-weighted imaging (T2WI-FS) sequence selectively suppresses fat signals, enhancing visualization of inflammatory edema in extraocular muscles. Previous studies suggest that signal intensity ratio (SIR) between extraocular muscles and reference tissues (ipsilateral brain white matter or temporalis muscle) may aid in staging TED activity. However, existing research mainly focuses on the most severely affected muscle, neglecting differential inflammatory involvement across the four rectus muscles. This study aims to compare the diagnostic efficacy of SIR of all rectus muscles in T2WI-FS for distinguishing active TED, evaluating SIR using both white matter and temporalis muscle as reference standards, and establishing potential diagnostic thresholds for differentiating active and inactive disease phases.

## Materials and methods

### Subjects

This study was approved by Affiliated Eye Hospital of Nanchang University. Written informed consent was obtained from each participant. From February 2024 to February 2025, 40 consecutive patients who were clinically diagnosed with TED were enrolled as TED group, except for one patient whose eye was a prosthetic eye. The 82% of the other patients (7/39) had symmetrical disease, which means their both eyes had the same degree of disease and the same CAS score, while there are 7 subjects have eyes in different disease states. A ratio of the total extraocular muscles area to orbit area (EMA: OA) was calculated for each orbit to distinguish whether it belonged to Type 1 TED patient (fat-predominant) or Type 2 TED patient (muscle-predominant). TED patients were classified as type I if their EMA: OA fell within the average control EMA: OA ± 2 SD; those exceeding this range were classified as type II (6). And healthy eyes of patients with unilateral orbital pathologies (tumors or trauma) during the same period were selected as the control group. This study was approved by the ethics committee (Approval code: YLS20240442) and adhered to the tenets of the Declaration of Helsinki.

### The inclusion criteria include

#### Inclusion criteria of TED group

1. All participants were between 18 and 65 years of age;
2. Meeting diagnostic criteria established by the European Group on Graves’ Orbitopathy (EUGOGO) (7);
3. Structural orbital MRI was scanned before treatment;
4. Image quality was adequate for further analysis;
5. No history of orbital radiotherapy or surgical interventions;
6. Absence of orbital pathologies from other causes (e.g., tumors, trauma, or infections);
7. EMA: OA > control Mean + 2 SD;
8. Capacity to complete clinical evaluations and MRI examinations.

#### Control group

1. Aged 18–65 years;
2. Healthy eyes of patients with unilateral orbital mass/trauma;
3. Unaffected healthy eye showing no pathological signs from the contralateral diseased eye;
4. No history of orbital radiotherapy or surgical interventions;
5. Capacity to complete clinical evaluations and MRI examinations.

### Clinical evaluation

Demographic data including age, gender, disease duration, and exophthalmos were collected. Disease duration was defined as the interval between TED symptom onset and MRI examination date. Exophthalmos was quantified using a Hertel exophthalmometer.

Disease activity staging was performed by 2 experienced ophthalmologists using the modified Clinical Activity Score (CAS) (8). The ophthalmologists performing the CAS assessments were blinded to the results of the MRI scans and the calculated SIR values at the time of scoring. Besides, to ensure standardized assessment, both ophthalmologists underwent a dedicated training session prior to the study beginning. This training involved reviewing the official CAS definitions, standardized photographic examples of each CAS component, and joint assessment of ambiguous cases to reach consensus on measuring method. Furthermore, the inter-observer agreement of the measuring outcome was calculated using Intraclass Correlation Coefficient (ICC) and showed excellent reliability (ICC = 0.91). The CAS evaluates seven parameters: (1) spontaneous eye pain, (2) eye pain upon eye movement, (3) eyelid erythema, (4) eyelid swelling, (5) conjunctival injection, (6) conjunctival redness, chemosis, and (7) caruncle swelling. Each parameter scores 1 point. A summed CAS score of ≥3 is considered as an active TED group, while others were assigned to the inactive TED group. The timing between CAS score and MRI scan for all patients was no more than 3 days.

### MRI protocol

Coronal orbital MRI was performed in each participant using a 3.0-T MRI scanner (Skyra, Siemens, Germany) with a 20-channel head coil. Patients were instructed to rest in supine position and close eyes to reduce motion-related errors. The signal intensities in the superior rectus (SR), inferior rectus (IR), lateral rectus (LR), and medial rectus (MR) were measured on the coronal fat-suppressed T2-weighted(T2WI-FS) image (repetition time = 4000 ms; echo time = 79 ms; slice thickness = 3 mm; FOV = 180 × 180 mm; voxel size = 0.5 × 0.5 mm).

### Image analysis

Two experienced ophthalmologists independently performed measurements while blinded to clinical information and study protocols. The observers demonstrated strong agreement, with intraclass correlation coefficients (ICC) exceeding 0.9 for all parameters. Their measurements were averaged for final analysis.

**Figure 1:**
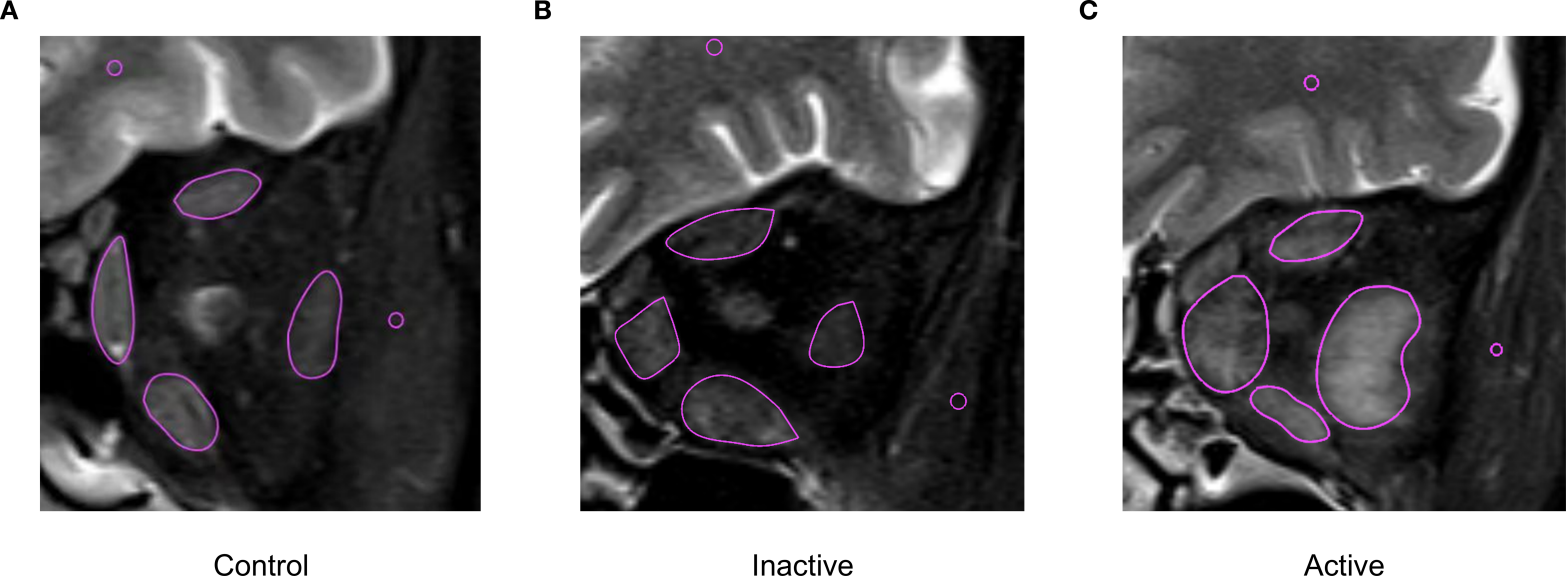
Measurement method of Signal intensity ratio (SIR). **(A)** Eye of control group **(B)** Eye of inactive TED group **(C)** Eye of active TED group.

On coronal T2WI-FS images, selecting a slice about 10mm away from the posterior wall of the eyeball (usually the third slice) and its adjacent anterior/posterior slices, regions of interest (ROIs) were manually drawn along the contours of four rectus muscles at three consecutive slices (9). Mean signal intensity was recorded for each rectus muscle. Simultaneously, standardized 1-mm² ROIs were placed on ipsilateral brain white matter and temporalis muscle at corresponding levels (**Figure 1**). Then SIR were calculated between each rectus muscle and reference tissues (brain white matter and temporalis muscle) in order to normalize the signal intensity of muscles: MR vs. white matter (SIR_M1), LR vs. white matter (SIR_L1), SR vs. white matter (SIR_S1), IR vs. white matter (SIR_I1), MR vs. temporalis muscle (SIR_M2), LR vs. temporalis muscle (SIR_L2), SR vs. temporalis muscle (SIR_S2), and IR vs. temporalis muscle (SIR_I2). The maximum SIR value across three slices was recorded as the final measurement.

### Statistical analysis

Statistical analysis was performed using SPSS 26.0. Statistical assumptions (normality, homogeneity of variance) were verified using Shapiro-Wilk and Levene’s tests respectively. Normally distributed continuous variables were expressed as means ± standard deviation (SD), while non-normally distributed data were reported as median [p25, p75]. Comparisons among three or more groups were analyzed using one-way ANOVA (for normal distributions) or the Kruskal-Wallis H test (for non-normal distributions). Gender differences were assessed with the Chi-square test. Receiver operating characteristic (ROC) curve analysis was employed to evaluate the predictive efficacy of SIR for disease activity and determine optimal cutoff values. Consistency of measurements between the two measurers was quantified using the intraclass correlation coefficient (ICC), with interpretation criteria: ICC <0.40 (poor), 0.40-0.60 (moderate), 0.61-0.80 (good), and ≥0.81 (excellent). A two-tailed P-value <0.05 was considered statistically significant for all analyses.

## Result

### Comparison of general data among three groups of patients

No statistically significant differences were observed in age or gender among the control group, inactive TED group, and active TED group (P > 0.05, **Table 1**). However, the disease duration of active TED patients was significantly shorter than that of inactive TED patients (4 [2, 9] months vs. 12 [6, 18] months, P = 0.000 < 0.05). While no significant difference in exophthalmos was found between active TED and inactive TED groups (P > 0.05), both groups exhibited significantly higher exophthalmos values compared to the control group (16 [15, 17] mm vs. 13.82 ± 3.18 mm; 17.25 [14, 19.25] mm vs. 13.82 ± 3.18 mm; all P = 0.000 < 0.05).

**Table 1:** The profile of three groups, control, inactive (CAS 1 or 2), and active (CAS > 3).

Parameters	Control(n=65)	TED		P-Value		
		Inactive(n=15)	Active(n=25)	Inactive vs. Control	Active vs. Control	Active vs. Inactive
Eyes	65	30	49			
Age	54.05 ± 13.65	55 ± 11.34	55.35 ± 13.020	0.74	0.597	0.908
Sex						
Male	30	14	19	0.963	0.431	0.490
Female	35	16	30			
Duration (m)	——	12 [6, 18]	4 [2, 9]	——	——	0.000
Exophthalmos (mm)	13.82 ± 3.1782	17.25 [14, 19.25]	16 [15, 17]	0.000	0.000	0.678
TRAb(IU/L)	——	1.35 [0.77, 2.19]	2.44 [1.10, 7.24]	——	——	0.095
TT3(nmol/L)	——	1.67 [1.50, 1.92]	1.73 [1.56, 2.33]	——	——	0.501
TT4(nmol/L)	——	125.29 [115.21, 147.62]	123.02 [107.52, 137.26]	——	——	0.319
FT3(pmol/L)	——	4.81 [4.58, 5.44]	5.01 [4.37, 5.36]	——	——	0.871
FT4(pmol/L)	——	11.43 [10.33, 12.67]	12.83 [10.57, 14.84]	——	——	0.016
TSH(μIU/L)	——	1.79 [0.55, 5.49]	1.01 [0.06, 3.36]	——	——	0.073
TGAb(IU/L)	——	32.12 [10.00, 200.62]	49.98 [10.00, 1590.12]	——	——	0.516
TMAb(IU/L)	——	17.42 [6.20, 59.36]	22.94 [3.83, 793.36]	——	——	0.533

Additionally, we compared thyroid-related parameters between inactive TED and active TED groups. Among these parameters, only free thyroxine (FT4) showed a significant intergroup difference (P = 0.016 < 0.05), while all other indices demonstrated no statistical significance.

### Comparison of SIR1 and SIR2 of four rectus muscles in three groups

**Figure 2:**
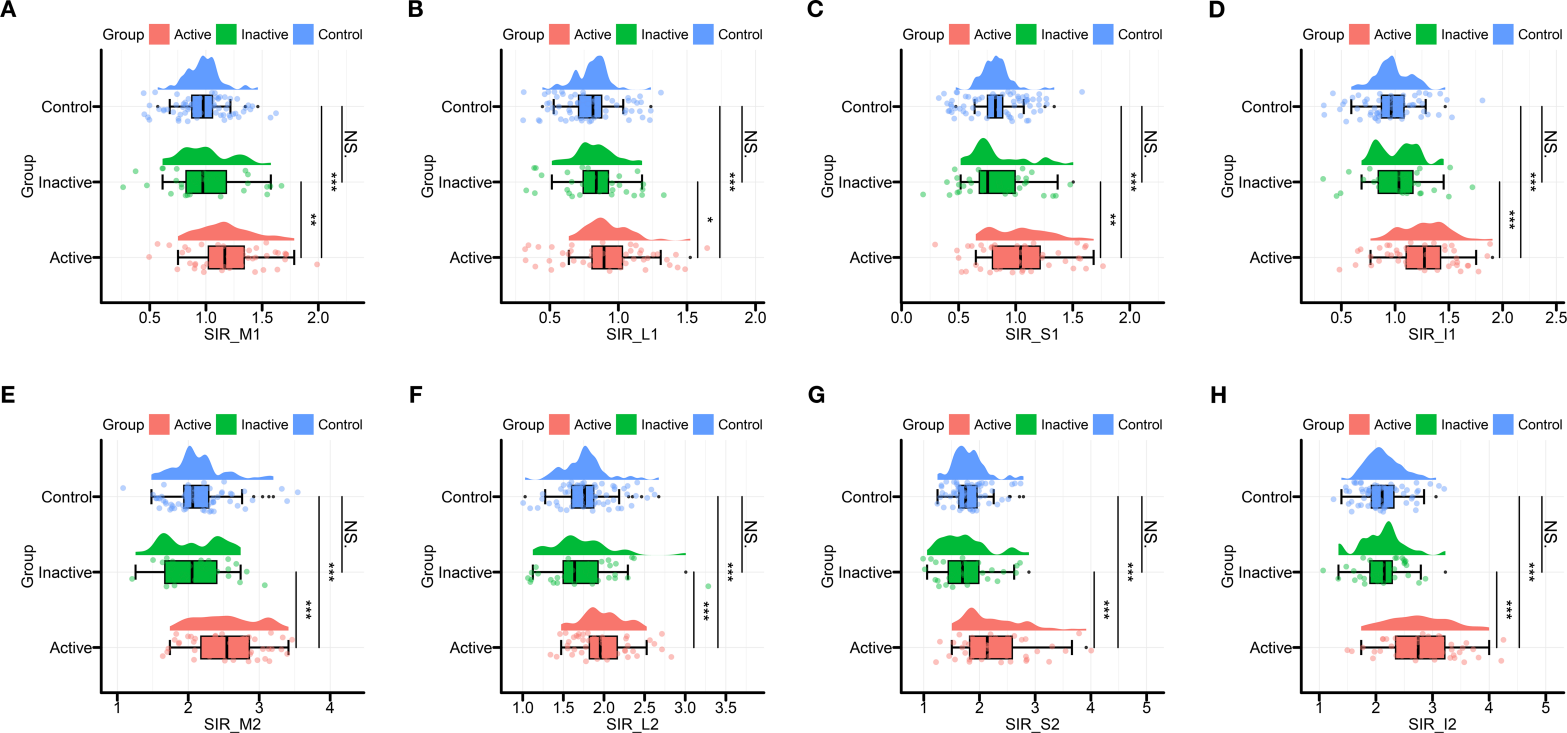
**(A)** Differences in signal intensity of medial rectus muscle/signal intensity of ipsilateral white matter among three groups (SIR_M1). **(B)** Differences in signal intensity of lateral rectus muscle/signal intensity of ipsilateral white matter among three groups (SIR_L1). **(C)** Differences in signal intensity of superior rectus muscle/signal intensity of ipsilateral white matter among three groups (SIR_S1). **(D)** Differences in signal intensity of inferior rectus muscle/signal intensity of ipsilateral white matter among three groups (SIR_I1). € Differences in signal intensity of medial rectus muscle/signal intensity of ipsilateral temporalis muscle among three groups (SIR_M2). **(F)** Differences in signal intensity of lateral rectus muscle/signal intensity of ipsilateral temporalis muscle among three groups (SIR_L2). **(G)** Differences in signal intensity of superior rectus muscle/signal intensity of ipsilateral temporalis muscle among three groups (SIR_S2). **(H)** Differences in signal intensity of inferior rectus muscle/signal intensity of ipsilateral temporalis muscle among three groups (SIR_I2). NS.p>0.05, *p<0.05, **p<0.01, ***p<0.001.

All four rectus muscles in the active TED group demonstrated significantly higher SIR (SIR1 and SIR2) compared to both inactive TED and control groups (P < 0.05), whereas no significant differences were observed between inactive TED and control groups (P > 0.05; **Table 2**, **Figure 2**).

**Table 2:** SIR1 and SIR2 of four extraocular muscles.

Parameters	Control(n=65)	TED		P-Value		
		Inactive(n=15)	Active(n=25)	Inactive vs. Control	Active vs. Control	Active vs. Inactive
SIR_M1	0.974 ± 0.155	1.001 ± 0.243	1.183 ± 0.2482	0.925	0.000	0.006
SIR_L1	0.800 ± 0.125	0.831 ± 0.155	0.921 ± 0.169	0.338	0.000	0.010
SIR_S1	0.826 ± 0.133	0.854 ± 0.260	1.048 ± 0.263	0.927	0.000	0.006
SIR_I1	0.977 ± 0.167	1.022 ± 0.199	1.277 ± 0.233	0.641	0.000	0.000
SIR_M2	2.142 ± 0.371	2.051 ± 0.421	2.548 ± 0.456	0.673	0.000	0.000
SIR_L2	1.755 ± 0.277	1.725 ± 0.389	1.980 ± 0.268	0.644	0.000	0.000
SIR_S2	1.815 ± 0.306	1.743 ± 0.455	2.265 ± 0.569	0.824	0.000	0.000
SIR_I2	2.138 ± 0.318	2.111 ± 0.411	2.775 ± 0.571	0.985	0.000	0.000

SIR1 represents signal ratio between extraocular muscles vs. white matter

SIR2 represents signal ratio between extraocular muscles vs. temporalis muscle

### The analysis of receiver operating characteristic curve

**Figure 3:**
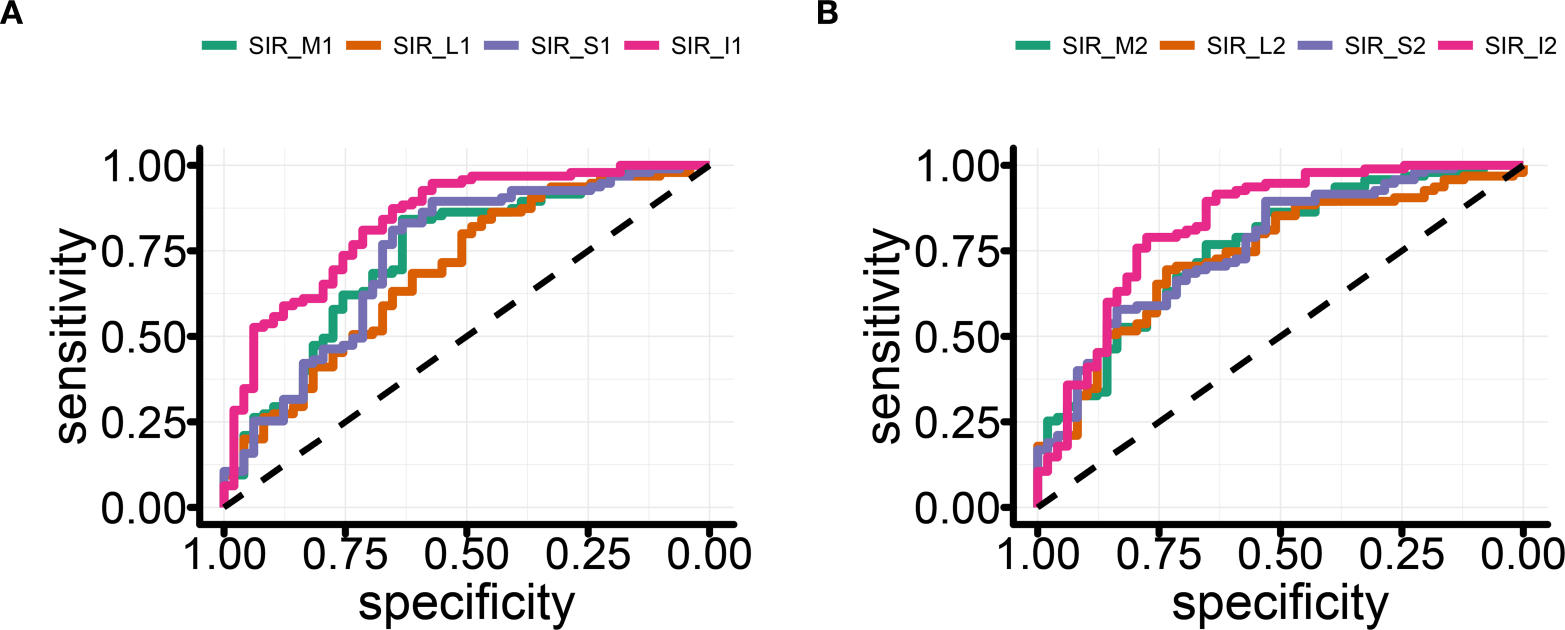
**(A)** SIR1 ROC curve distinguishing active TED group from control group + inactive TED group. **(B)** SIR2 ROC curve distinguishing active TED group from control group + inactive TED group.

Receiver operating characteristic (ROC) curves for the four rectus muscles can help us intuitively compare the effectiveness of each muscle in distinguishing TED activity (**Figure 2**). SIR1 values showed good predictive efficacy for distinguishing active and inactive phases, with the highest area under the curve (AUC) observed in the IR (AUC = 0.837), followed by the MR and SR (both AUC = 0.739), and the lowest in the LR (AUC = 0.691; **Figure 3**).

Interestingly, SIR2 generally exhibited superior diagnostic performance compared to SIR1 (medial: 0.759 vs. 0.739; superior: 0.763 vs. 0.739; lateral: 0.740 vs. 0.691). However, the IR uniquely showed higher AUC with SIR1 than SIR2 (0.829 vs. 0.837). Among SIR2 measurements, diagnostic efficacy ranked as follows: inferior > superior > medial > lateral (**Figure 3**). Specific cutoff values, P-values, and 95% confidence intervals are detailed in **Tables 3–1** and **Tables 3–2**.

**Table 3–1:** ROC curve parameters of SIR1 (four rectus muscles).

Parameters	Cutoff	Sensitivity	Specificity	Youden Index	AUC	P-value	95% CI
SIR_I1	1.206	0.653	0.874	0.527	0.837	0.000	0.766-0.907
SIR_M1	1.103	0.633	0.842	0.475	0.739	0.000	0.651-0.827
SIR_S1	1.012	0.571	0.895	0.466	0.739	0.000	0.649-0.829
SIR_L1	0.918	0.449	0.863	0.312	0.691	0.000	0.599-0.784

**Table 3–2:** ROC curve parameters of SIR2 (four rectus muscles).

Parameters	Cutoff	Sensitivity	Specificity	Youden Index	AUC	P-value	95% CI
SIR_I2	2.332	0.776	0.789	0.565	0.829	0.000	0.753-0.905
SIR_S2	2.119	0.531	0.895	0.426	0.763	0.000	0.681-0.845
SIR_M2	2.344	0.653	0.768	0.421	0.759	0.000	0.676-0.841
SIR_L2	1.843	0.735	0.695	0.43	0.740	0.000	0.656-0.824

### Distribution trend of SIR value of each muscle

Our data shows that in both the control group and the TED group, the highest SIR values (including SIR1 and SIR2) among the four rectus muscles typically occurred in either the IR or MR, with the IR being more frequent (48.1% vs. 34.18%; 55.38% vs. 44.62%; 46.84% vs. 36.71%; 55.38% vs. 44.62%). The SR followed as the next most common, while the LR had the lowest occurrence of peak SIR values. We also observed that in the control group, the distribution pattern of the highest SIR1 values across the muscles matched that of SIR2, and a similar pattern was seen in the TED group as well. Additionally, within the control group, the highest SIR values never occurred in the LR or SR, as detailed in **Table 4**.

**Table 4:** Muscle distribution proportion with maximum SIR.

Group	SIR_M1	SIR_L1	SIR_S1	SIR_I1	SIR_M2	SIR_L2	SIR_S2	SIR_I2
Total	56(38.89%)	2(1.39%)	12(8.33%)	74(51.39%)	58(40.28%)	2(1.39%)	11 (7.64%)	73(50.69%)
Control	29(44.62%)	0	0	36(55.38%)	29(44.62%)	0	0	36(55.38%)
TED	27(34.18%)	2(2.53%)	12(15.19%)	38(48.1%)	29(36.71%)	2(2.53%)	11 (13.92%)	37(46.84%)

### A novel approach for TED activity staging

The above findings were established based on CAS-based staging of TED activity. However, considering the inherent limitations of CAS scoring, we propose a conceptual framework that may inform future clinical and research practices. Taking SIR1 of IR as an example, comparative analysis of 95% confidence intervals (CIs) and ROC cutoff values across groups revealed that there were differences in the distribution of the 95% confidence intervals in the control group (0.936-1.019), the inactive TED group (0.948-1.097) and the active TED group (1.210-1.344), and the ROC cutoff value was close to the lower bounds of the active period. While partial overlap existed between the CIs of the control and inactive TED groups (with the lower limits of these two groups remained comparable), the upper limit of the inactive TED group being 7.6% higher than controls. This overlapping pattern suggests that some patients classified as CAS <3 (“inactive”) may harbor subclinical orbital inflammation undetectable by CAS due to its limited sensitivity for deep orbital involvement. Such cases could explain the clinical paradox of disease progression in ostensibly “inactive” patients.

**Figure 4:**
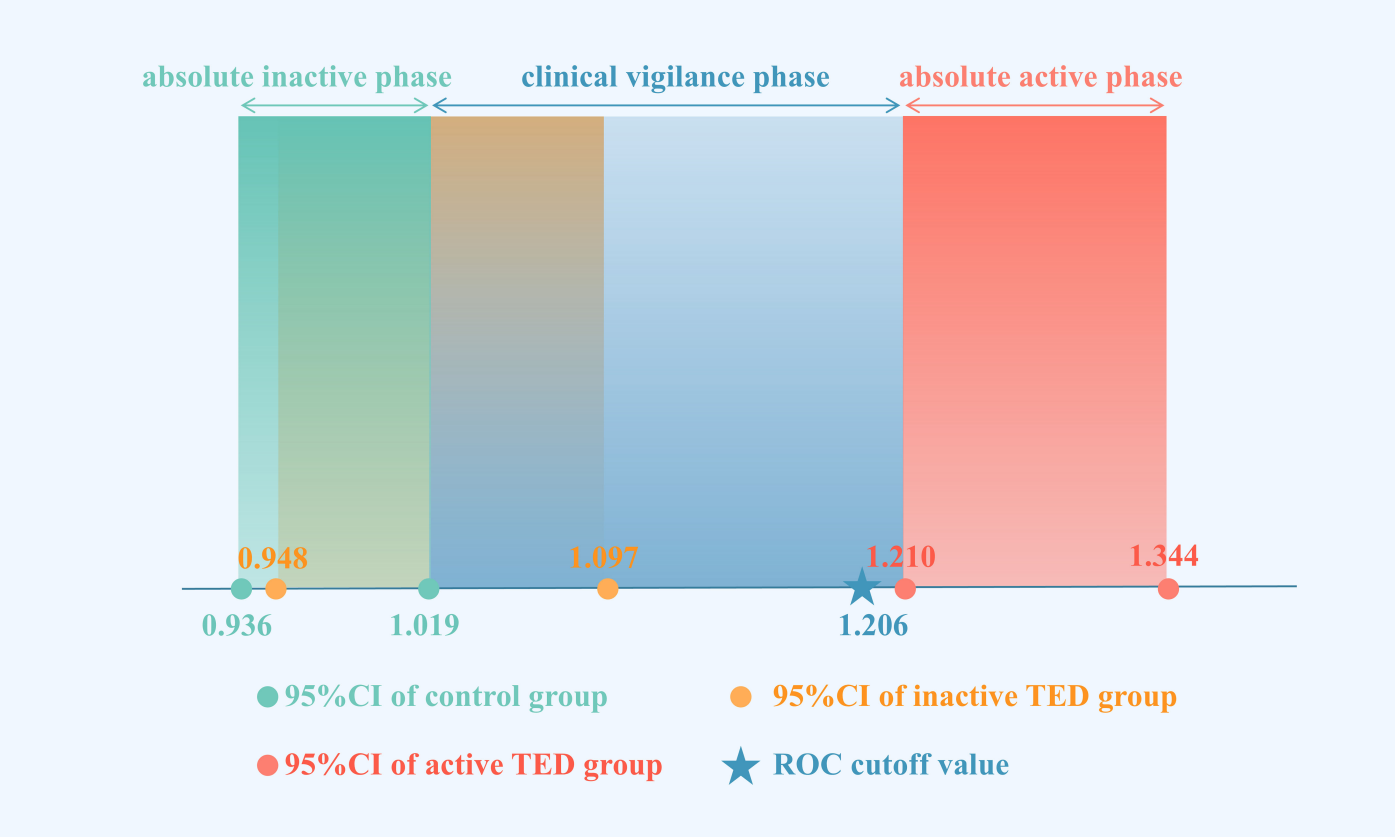
95% confidence intervals and ROC cutoff values for SIR_I1 of control group, inactive TED group, and active TED group. (Green dots with corresponding rectangles represent the lower/upper bounds and confidence domains of the control group’s 95% CI. Yellow dots with corresponding rectangles represent the lower/upper bounds and confidence domains of the inactive TED group’s 95% CI. Blue stars represents the diagnostic cutoff value of the ROC curve, with blue rectangular zones marking the transitional range between the control group’s upper CI limit and active TED group’s lower CI limit. TED activity staging using MRI-derived SIR criteria: green zone = absolute inactive phase; blue zone = clinical vigilance phase; red zone = absolute active phase.).

Based on these observations, we propose redefining staging criteria as follows: The control group’s CI (0.936-1.019) may represent an absolute inactive phase, while the active TED group’s CI (1.210-1.344) defines an absolute active phase. Patients falling within the intermediate range (1.019-1.210, currently classified as CAS <3) can define a clinical vigilance phase which would constitute a transitional cohort requiring heightened clinical vigilance (**Figure 4**). For this group, comprehensive evaluation integrating thyroid function tests, thyrotropin receptor antibody levels, and systemic inflammatory markers is recommended to identify early inflammatory activity. Proactive management in these patients may help delay inflammatory progression and reduce irreversible complications.

## Discussion

The pathophysiological hallmark of active TED involves intensified inflammatory responses in orbital connective tissues, whereas structural changes in anterior orbital components often exhibit delayed manifestation. Consequently, reliance solely on the Clinical Activity Score (CAS) may delay early diagnosis of active disease. Timely identification of inflammatory activity is clinically critical for guiding immunosuppressive therapy and preventing irreversible complications including extraocular muscle fibrosis and compressive optic neuropathy (4). T2WI-FS, the focus of this study, specifically detects edema-associated signal alterations indicative of inflammation. Although emerging MRI sequences such as T1 mapping, T2 mapping, Dixon, and diffusion-weighted imaging (DWI) are gaining attention in TED differential diagnosis, T2WI-FS demonstrates superior clinical applicability and ease of implementation due to its widespread availability, cost-effectiveness, and mature image interpretation (10–12). While magnetic field inhomogeneity may affect precision, we can improve accuracy through standardizing measurements such as using the temporalis muscle and white matter as references. Additionally, SIR addresses the limitations of subjective methods like CAS by providing an objective quantitative measure. It visualizes posterior orbital involvement and reflects inflammatory changes through continuous values – elevated SIR indicates active edema, while reduced SIR suggests fibrotic collagen deposition. Although some studies use DWI- apparent diffusion coefficient (ADC) to assess retrobulbar inflammation, it requires more complex calculations. In contrast, SIR only requires measuring two ROIs for ratio calculation, making it more practical for wider adoption (13).

Through comparative analysis, it was found that there was no significant difference in the SIR1 and SIR2 of the four extraocular muscles between the inactive TED group and the control group, indicating the absence of marked inflammatory activity in inactive patients and consequently explaining the suboptimal therapeutic response to anti-inflammatory interventions during this phase. In contrast, significantly elevated SIR1 and SIR2 values across all four extraocular muscles in the active TED group compared to the inactive group demonstrated noticeable inflammatory edema, supporting the clinical reason for initiating anti-inflammatory treatment in active TED patients. These findings align with previous research conclusions (14–16). However, discrepancies exist with Politi et al.’s study, which reported significant SIR differences between controls and inactive TED patients, potentially attributable to their smaller control group size and baseline characteristic differences among study participants (17). Further multi-center studies with standardized protocols are warranted to reconcile these discrepancies.

ROC curve analysis was employed to compare the diagnostic efficacy of four extraocular muscles in distinguishing TED phases. Notably, the IR demonstrated optimal discriminative capacity (SIR1 AUC = 0.837, SIR2 AUC = 0.829), potentially attributable to its fibroblasts’ high expression of thyroid-stimulating hormone receptor (TSHR) and insulin-like growth factor-1 receptor (IGF-1R) (1). In contrast, the LR exhibited the lowest detection sensitivity, possibly due to distinct neurovascular supply and immune microenvironment characteristics. However, different conclusions appeared in SIR1 and SIR2 in the identification efficiency of the MR and SR, and this difference also existed in the study of Li et al. (18).These variations may arise from measurement challenges, including the small anatomical size of the MR and occasional difficulty in differentiating the SR from the levator palpebrae superioris muscle. These findings generally align with the previously recognized order of extraocular muscle involvement, similar to the results of some previous studies (7, 18, 19). Therefore, the IR is recommended as the primary anatomical site for TED activity assessment.

We must acknowledge that due to individual variations, disease progression patterns differ among patients, leading to differences in SIR changes across muscles. Although the IR is widely recognized in most studies as having the best staging capability (11), this does not mean it exhibits the most severe inflammatory edema, nor does it imply that its SIR value is necessarily the highest. In all the orbits we evaluated, the IR indeed showed the highest SIR among the four rectus muscles in the largest proportion of cases (51.39%, 50.69%), but the MR also accounted for a considerable share (38.89%, 40.28%). Even in cases where the IR does not show the highest SIR in individual patients, its SIR may still be the most stable and discriminative overall. Therefore, we recommend prioritizing the clinical assessments of the IR, while other muscles such as the MR should also be evaluated in atypical cases.

Additionally, some studies have found that a small proportion of TED patients with low CAS scores may only exhibit involvement of the SR/LSP muscle complex on MRI (20), and they may present with eyelid retraction as the initial symptom. Such involvement of the SR/LSP often occurs earlier than in other muscles (such as the IR), yet it tends to be less pronounced—for instance, showing less thickening and relatively mild abnormal SIR value—making it easily overlooked or underestimated. Therefore, the timing of MRI examination is crucial. If imaging is performed during this early stage when SR/LSP involvement dominates, but muscles like the IR are used for diagnosis and staging, the results may be compromised. In our dataset, the SR was indeed observed to be the third most frequent muscle to show the highest SIR value—after the IR and MR—it should be noted that our measurements included the SR/levator palpebrae superioris complex. Moreover, none of the healthy controls exhibited the highest SIR in this muscle, supporting its specificity in TED assessment. However, analysis of our current data did not reveal a clear or consistent pattern of early SR/LSP involvement. For example, among individuals with CAS < 3, only 11.58% exhibited SIR1 and SIR2 values above the cutoff, and only four cases showed the highest values in both SIR_S1 and SIR_S2, which is inconsistent with our clinical understanding. This discrepancy may be attributed to the small sample size and the fact that patients visiting the hospital often present at moderate to severe disease stages, resulting in insufficient inclusion of suitable early-stage TED cases and introducing certain selection biases. The conclusion that “the IR is better” may be more applicable in relatively overt phases of the disease, whereas different considerations might be needed for early-stage subgroups. In future research and clinical practice, greater attention should be paid to MRI signals of the SR/LSP in suspected early TED cases or patients with low CAS scores.

This study conducted the first systematic comparison of diagnostic efficacy between SIR1 and SIR2, revealing SIR2’s superior discrimination capacity. However, Pająk et al. (21) reported that white matter serves as a more reliable and practical normalization reference than the temporalis muscle. This discrepancy may be attributed to the larger and more homogeneous signal regions of white matter in MRI, combined with their younger cohort having lower prevalence of cerebrovascular diseases or hypertension that could alter white matter signals (22). Nevertheless, the drawbacks of their findings is evident due to a small sample size (7 healthy individuals) and absence of TED patient data. Additionally, while the lacrimal gland has gained recent attention as a potential signal measurement site, it was excluded from this study due to insufficient anatomical recognition on T2WI-FS sequences. Further studies integrating reference tissues above in larger TED cohorts are warranted to validate these findings.

In contrast to prior studies focusing solely on the most severely affected extraocular muscle’s SIR, this investigation systematically measured SIR in all four rectus muscles, yielding more comprehensive conclusions and enabling comparative analysis of inter-muscular signal variations. Additionally, our methodology of delineating the entire muscle cross-section as ROIs demonstrates improved anatomical precision compared to the conventional 1mm² point-ROI approach, thereby minimizing sampling bias. Beyond mean muscle SIR, we also evaluated maximum signal intensity ratio (SIRmax) across the four muscles. While intergroup SIRmax differences were observed, ROC analysis demonstrated AUC values below 0.4 (AUC <0.4), indicating this parameter lacks diagnostic utility for TED activity differentiation.

While addressing the early detection limitations of the CAS, this study innovatively established SIR-based early identification ranges (SIR1: 1.019-1.206; SIR2: 2.217-2.332) derived from 95% confidence intervals. Actually, increasing number of studies have revealed that CAS <3 exhibits activity, suggesting the need to establish MRI baseline criteria using healthy control groups. For example, existing research has indicated that when CAS are low, combining MRI can improve sensitivity in detecting inflammatory activity. It is recommended that even if only one CAS parameter is positive, orbital MRI should still be performed (23). Another study on imaging biomarkers in a transitional range applied a non-EPI-DWI sequence, demonstrating that this technique can guide clinical decision-making in patients with moderate disease activity (CAS 1–3), a finding that aligns closely with the methodology used in our study (24).

Notably, a diagnostic discontinuity emerged between the upper limit of the inactive TED group and the lower limit of the active TED group, primarily attributed to potential confounding biases. These include the selection favors hospitalized patients where most patients in the active TED group are patients with significant inflammation, whereas the inactive TED group largely comprised fibrotic end-stage patients requiring orbital decompression surgery. Additionally, this gap may be potentially compounded by the rapid progression of TED-associated inflammation resulting in limited transitional cases within the study population.

The study found that active patients had significantly higher free thyroxine [FT4] levels compared to those in the inactive stage, while other parameters including thyroid stimulating receptor antibody [TRAb], free triiodothyronine [FT3], total thyroxine [TT4], thyrotropin [TSH], etc. showed no significant differences between groups. This finding contradicts previous reports (15, 25, 26). We speculate this apparent discrepancy may be related to the fact that some participants had received prior immunotherapy due to chronic thyroid dysfunction. Therefore, although this observation supports the potential role of FT4 in assessing TED activity, its biological relevance requires further interpretation considering patients’ treatment history. Additionally, while thyroid-stimulating immunoglobulin (TSI) has demonstrated diagnostic value in certain studies (27, 28), the current research did not include relevant test items for comparison due to the absence of corresponding test data.

This research demonstrated that the SIR from T2WI-FS sequences serves as a reliable predictor for TED activity. Both SIR1 and SIR2 showed good predictive performance, with the IR exhibiting optimal diagnostic efficacy. Cutoff values of SIR_I1 >1.206 or SIR_I2 >2.332 effectively identified active TED, while SIR2 demonstrated enhanced discriminatory potential compared to SIR1. Notably, the transitional range between the upper control limit and lower active TED limit–clinical vigilance phase–may help detect possible inflammatory activity, potentially guiding clinical decision-making.

However, the findings should be interpreted with consideration of the study’s limitations. There is conception that TED manifests as two predominant phenotypes: Type I (fat-predominant) and Type II (muscle-predominant) (6). Our cohort specifically enrolled patients with radiologically confirmed extraocular muscles hypertrophy, which aligns with the classic Type II phenotype. However, we recognize that our findings may not apply to Type I TED patients. Although inflammation and expansion of the orbital fat are common in Type I patients, the extraocular muscles are usually not involved. Therefore, extraocular muscles status on MRI does not necessarily reflect genuine orbital inflammation, making the extraocular muscles’ SIR an unreliable biomarker in this subgroup. Instead, SIR of fat-to-white matter or volume of fat may prove more relevant biomarkers in Type I TED. In the future, prospective comparison of SIR between TED subtypes using consensus phenotyping criteria is necessary, and the situation in hybrid models combining extraocular muscle-SIR and fat-SIR needs to be explored also. Additionally, this is a single-center retrospective study including a relatively small sample size with geographic bias (predominance of southern Chinese participants) and incomplete clinical records that precluded analysis of confounding factors such as smoking and BMI (29).

## Data Availability

The raw data supporting the conclusions of this article will be made available by the authors, without undue reservation.
